# Malnutrition Severity Drives Mortality in Geriatric Heart Failure: A Multicenter Extreme Gradient Boosting Analysis

**DOI:** 10.31083/RCM48595

**Published:** 2026-05-21

**Authors:** Yiming Chen, Min He, Mengyu He, Ziting Yuan, Xiao Chen

**Affiliations:** ^1^Department of Geriatrics, The First Affiliated Hospital of Bengbu Medical University, 233004 Bengbu, Anhui, China; ^2^Department of Emergency, The First Affiliated Hospital of Bengbu Medical University, 233004 Bengbu, Anhui, China; ^3^Department of Nursing, The First Affiliated Hospital of Bengbu Medical University, 233004 Bengbu, Anhui, China

**Keywords:** heart failure, malnutrition, elderly patients, machine learning, SHAP

## Abstract

**Background:**

Heart failure (HF) and malnutrition frequently coexist in older patients (≥65 years) and are major determinants of in-hospital mortality. However, predictive models specifically addressing this high-risk population remain limited. Therefore, this study aimed to develop and validate a personalized machine learning model to assess key risk factors.

**Methods:**

This study was a multicenter retrospective investigation that collected clinical data from older patients with HF and malnutrition admitted to two Chinese tertiary hospitals. Key predictors were selected using least absolute shrinkage and selection operator (LASSO) regression, followed by development of an extreme gradient boosting (XGBoost) model. Model performance was assessed using receiver operating characteristic (ROC) curve analysis, accuracy, sensitivity, specificity, and F1 score. Shapley additive explanation (SHAP) analysis was applied to provide interpretable feature importance. Moreover, the robustness of the model was externally validated in an independent cohort.

**Results:**

The final analysis included 1080 older patients with HF and malnutrition, among whom 244 experienced in-hospital mortality, yielding an in-hospital mortality rate of 22.6%. The XGBoost model achieved high area under the curve (AUC) values (training: 0.979, 95% confidence interval (CI): 0.969–0.990; validation: 0.890, 95% CI: 0.844–0.937; test: 0.936, 95% CI: 0.899–0.974). SHAP analysis highlighted the Geriatric Nutritional Risk Index (GNRI) as the primary predictive factor, with secondary contributions from inflammatory profiles and traditional cardiorenal and electrolyte markers.

**Conclusions:**

The constructed XGBoost model demonstrated robust predictive performance. The SHAP analysis provided a clear visualization of key risk factors, thereby providing a valuable reference for clinical risk assessment.

## 1. Background 

Heart failure (HF) is often the final common pathway for primary cardiovascular diseases, characterized by both high morbidity and mortality rates [1]. HF is a major global health concern, with an increasing prevalence driven by ageing populations, better treatment outcomes and improved survival [2]. Chronic diseases typically represent a state of persistent inflammation, marked by increased catabolism and reduced anabolism [3, 4]. The phenomenon is particularly pronounced in patients with HF [5]. Due to inadequate recognition and lack of nutritional interventions, elderly patients with HF frequently develop comorbid malnutrition in clinical practice [6].

Malnutrition has been identified as an independent risk factor for mortality in patients with HF [2, 7, 8]. Early screening and intervention are crucial for HF patients with malnutrition. However, the new major HF guidelines lack specific nutrition recommendations [1]. There are many types of nutritional screening and assessment tools designed for various types of patients, such as surgical, cancer, and chronic disease patients [9]. The primary nutritional screening and assessment tools for heart failure patients include the mini nutritional assessment (MNA), MNA short form (MNA-SF), geriatric nutritional risk index (GNRI), controlling nutritional status (COUNT), and nutritional risk screening (NRS) [8, 9]. MNA and GNRI hold particular predictive value for poor prognosis in HF patients [2, 8]. GNRI was ultimately used to assess patients’ nutritional status in this study, as its applicable population aligns with our research cohort and due to the data selection limitations inherent in retrospective studies.

Biomarkers play a pivotal role in prognostic evaluation of HF patients [3]. Some traditional biomarkers like N-terminal pro-B-type natriuretic peptide (NT-proBNP), absolute neutrophil count (ANC) and absolute lymphocyte count (ALC) demonstrate super predictive power for adverse outcomes [10]. Meanwhile, recent studies have validated the prognostic capability of novel inflammatory markers like neutrophil to lymphocyte ratio (NLR) and platelet to lymphocyte ratio (PLR) [10, 11, 12]. It is indicated that multi-marker models may have better risk prediction [13], we comprehensively incorporated both tradition and novel biomarkers in this study.

Machine learning (ML) algorithms have been gradually applied to the prediction of cardiovascular diseases [14]. Several ML predictive models have been built for assessing mortality in patients with HF [15]. Nevertheless, there remains a paucity of clinical prediction models specifically designed for elderly patients with concurrent HF and malnutrition. A 2024 study developed multiple clinical prediction models for cardiovascular diseases based on biomarkers, which showed that the Extreme Gradient Boosting (XGBoost) model demonstrated the best predictive performance (AUC = 0.9921) [16]. What’s more, the study by Li *et al*. [17], based on 2798 HF patients, compared the predictive performance of four models for in-hospital mortality, with results demonstrating XGBoost as the optimal predictive model (AUC = 0.824).

In summary, this study evaluated nutritional status in elderly HF patients using GNRI and incorporated both traditional and novel biomarkers to develop an XGBoost clinical prediction model for mortality risk assessment in elderly HF patients with malnutrition. The findings provide a foundation for developing targeted therapeutic strategies.

## 2. Methods

### 2.1 Study Population and Data Source

**Fig. 1.**
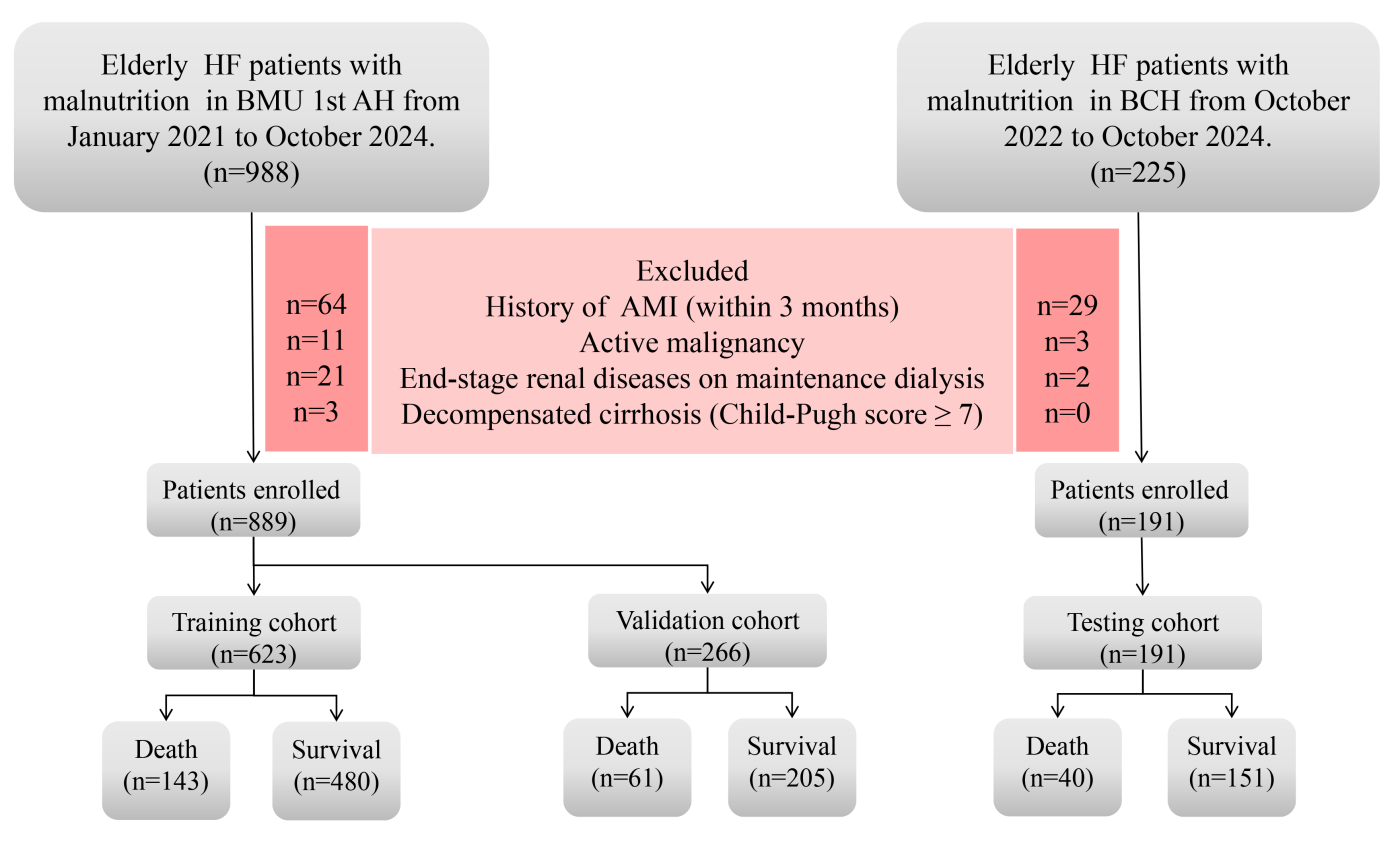
**A flowchart illustrating the data screening process of this study**. HF, heart failure; AMI, acute myocardial infarction; BMU 1st AH, first affiliated hospital of bengbu medical university; BCH, bengbu central hospital.

We collected the data of elderly HF patients with malnutrition (female or male ≥65 years) who were admitted to Bengbu Central Hospital (BCH) between October 2022 to October 2024 and the first affiliated hospital of Bengbu medical university (BMU 1st AH) between January 2021 to October 2024. The inclusion criteria were as follows: (1) patients with a confirmed diagnosis of HF; (2) patients had presence of nutritional impairment evidenced by the GNRI (score ≤98). The exclusion criteria were as follows: (1) history of acute myocardial infarction (AMI) (within 3 months); (2) active malignancy; (3) end-stage renal diseases on maintenance dialysis; (4) decompensated cirrhosis (Child-Pugh score ≥7). Following screening, 889 patients from BMU 1st AH and 191 patients from BCH were analyzed (Fig. 1). Laboratory data were collected within 24 hours of admission for non-transferred patients, or within 24 hours post-transfer for those moved between departments. The primary endpoint was all-cause in-hospital mortality, assessed as a binary outcome (survival or death) at discharge. This study received ethical approval from the ethics committee of the first affiliated hospital of Bengbu medical university, China (No.2025-551), and was granted an exemption by Bengbu central hospital. Due to the retrospective observational nature of this study, the requirement for informed consent was waived.

### 2.2 Data Preprocessing and Feature Selection

Guided by the underlying causes of HF in geriatric patients, established clinical practice, and contemporary literature, we selected 62 variables for this study. The primary aim of this study was to develop a mortality prediction model. Accordingly, we collected binary outcome data (death or survival) for all patients. In addition, we collected demographic characteristics including sex, age, weight, height and body mass index (BMI) was calculated for analytical purposes. Comorbidities such as diabetes mellitus (DM), coronary artery disease (CAD), arrhythmia, valvular heart disease (VHD), hypertension (HTN), pulmonary diseases, urinary tract infection (UTI), cerebral infarction and so on. Our study evaluated patients’ nutritional status using the GNRI, calculated as:

GNRI = [1.489 × albumin (g/dL)] + [41.7 × (current weight / ideal weight)].

Male ideal weight (kg) = height (cm) – 100 – [height (cm) – 150] / 4.

Female ideal weight (kg) = height (cm) – 100 – [height (cm) – 150] / 2.5.

We assessed nutritional risk using the GNRI, including all patients with a score ≤98 (indicative of malnutrition risk) and recording their specific values.

Therefore, we recorded serum albumin (ALB) levels at both admission and discharge, along with the total dosage of human albumin administered during hospitalization. NT-proBNP and left ventricular ejection fraction (LVEF) were systematically included in our study which as gold-standard diagnostic markers for HF. Additionally, we collected the following admission laboratory parameters: white blood cell (WBC), neutrophil percentage (NEUTperc), lymphocyte percentage (LYperc), hemoglobin (Hb), platelet (PLT), creatinine (Cr), blood urea nitrogen (BUN), aspartate aminotransferase (AST), alanine aminotransferase (ALT), cholinesterase (AchE), electrolyte levels, C-reactive protein (CRP), total cholesterol (TC), triglyceride (TG), low-density lipoprotein cholesterol (LDL-C), high-density lipoprotein cholesterol (HDL-C), cardiac troponin I (cTnI), creatine kinase-MB (CK-MB) and so on. And we calculated six novel inflammatory markers for model construction: NLR, PLR, systemic immune inflammation index (SII), neutrophil percentage to albumin ratio (NPAR), lymphocyte to C-reactive protein ratio (LCR), Platelet to high density lipoprotein cholesterol ratio (PHR).

(1) NLR = Neutrophil count (×10^9^/L) / Lymphocyte count (×10^9^/L).

(2) PLR = Platelet count (×10^9^/L) / Lymphocyte count (×10^9^/L).

(3) SII = (Platelet count × Neutrophil count) / Lymphocyte count (all (×10^9^/L)).

(4) NPAR = Neutrophil percentage (%) / Serum albumin (g/dL).

(5) LCR = Lymphocyte count (×10^9^/L) / C-reactive protein (mg/L).

(6) PHR = Platelet count (×10^9^/L) / High-density lipoprotein cholesterol (mmol/L).

Due to the inability to perform echocardiography in some acutely ill HF patients during hospitalization, the LVEF data had a significant missing rate of 11.12%. To ensure the robustness of statistical inferences, multiple imputation was performed using the mice package in R to generate 5 complete datasets. For the purpose of describing baseline characteristics in Table 1, the first imputed database was used, as is the conventional practice for descriptive statistics. And due to the strong correlations among some variables, the study employed the least absolute shrinkage and selection operator (LASSO) technique to pick out key clinical factors, while simultaneously discarding extraneous data.

**Table 1.** **Baseline characteristics of elderly patients with heart failure and malnutrition**.

Variables		The study cohort (training and validation, n = 889), n (%)	Group, n (%)			Testing cohort (n = 191), n (%)
			Survival (n = 685)	Death (n = 204)	*p*	
Arrhythmia, n (%)					0.020	
	No	369 (41.51)	270 (39.42)	99 (48.53)		84 (43.98)
	Yes	520 (58.49)	415 (60.58)	105 (51.47)		107 (56.02)
Valvular disease (%)					0.377	
	No	754 (84.81)	577 (84.23)	177 (86.76)		172 (90.05)
	Yes	135 (15.19)	108 (15.77)	27 (13.24)		19 (9.95)
Cardiomyopathy (%)					0.389	
	No	815 (91.68)	625 (91.24)	190 (93.14)		178 (93.19)
	Yes	74 (8.32)	60 (8.76)	14 (6.86)		13 (6.81)
CAD (%)					0.553	
	No	325 (36.56)	254 (37.08)	71 (34.80)		61 (31.94)
	Yes	564 (63.44)	431 (62.92)	133 (65.20)		130 (68.06)
Pulmonary diseases (%)					0.004	
	No	735 (82.68)	580 (84.67)	155 (75.98)		139 (72.78)
	Yes	154 (17.32)	105 (15.33)	49 (24.02)		52 (27.22)
HTN (%)					0.373	
	No	452 (50.85)	356 (51.97)	96 (47.06)		82 (42.93)
	Stage 1	22 (2.47)	17 (2.48)	5 (2.45)		4 (2.09)
	Stage 2	116 (13.05)	92 (13.43)	24 (11.76)		33 (17.28)
	Stage 3	299 (33.63)	220 (32.12)	79 (38.73)		72 (37.70)
DM (%)					0.405	
	No	652 (73.34)	507 (74.01)	145 (71.08)		151 (79.06)
	Yes	237 (26.65)	178 (25.99)	59 (28.92)		40 (20.94)
Pulmonary infection (%)					<0.001	
	No	443 (49.83)	384 (56.06)	59 (28.92)		83 (43.46)
	Yes	446 (50.17)	301 (43.94)	145 (71.08)		108 (56.54)
Cerebral infarction (%)					0.068	
	No	703 (79.08)	551 (80.44)	152 (74.51)		145 (75.92)
	Yes	186 (20.92)	134 (19.56)	52 (25.49)		46 (24.08)
EN (%)					<0.001	
	No	820 (92.24)	655 (95.62)	165 (80.88)		NA
	Yes	69 (7.76)	30 (4.38)	39 (19.12)		NA
ICU/CCU (%)					<0.001	
	No	530 (59.62)	445 (64.96)	85 (41.67)		126 (65.97)
	Yes	359 (40.38)	240 (35.04)	119 (58.33)		65 (34.03)
Hypokalemia (%)					0.811	
	No	681 (76.60)	526 (76.79)	155 (75.98)		146 (76.44)
	Yes	208 (23.40)	159 (23.21)	49 (24.02)		45 (23.56)
Hyponatremia (%)					<0.001	
	No	671 (75.48)	547 (79.85)	124 (60.78)		158 (82.72)
	Yes	218 (24.52)	138 (20.15)	80 (39.22)		33 (17.28)
UTI (%)					0.043	
	No	715 (80.43)	561 (81.90)	154 (75.49)		147 (76.96)
	Yes	174 (19.57)	124 (18.10)	50 (24.51)		44 (23.04)
LVEF (median (IQR))		42 (40, 45)	42 (39, 47)	41 (40, 42)	0.453	43 (39, 49.5)
Age (median (IQR))		78 (72, 83)	78 (72, 83)	79 (73, 85)	0.013	79 (72, 86)
BMI (median (IQR))		21.88 (19.53, 24.77)	22.22 (20.03, 24.97)	19.92 (18.32, 23.44)	<0.001	22.12 (19.93, 24.33)
GNRI (median (IQR))		92.11 (87.26, 95.79)	93.37 (89.44, 96.5)	85.68 (80.01, 90.39)	<0.001	93.07 (87.83, 96.64)
WBC (median (IQR))		7.09 (5.41, 10.01)	6.54 (5.09, 8.75)	10.46 (7.69, 15.2)	<0.001	6.45 (5.27, 8.94)
ANC (median (IQR))		5.29 (3.65, 8.22)	4.7 (3.38, 6.74)	9.06 (6.33, 13.45)	<0.001	4.64 (3.56, 6.95)
ALC (median (IQR))		0.99 (0.7, 1.39)	1.1 (0.79, 1.48)	0.67 (0.4, 1.02)	<0.001	0.98 (0.69, 1.35)
AMC (median (IQR))		0.51 (0.36, 0.69)	0.49 (0.37, 0.66)	0.56 (0.35, 0.81)	0.014	0.49 (0.36, 0.7)
NEUT% (median (IQR))		76 (66.6, 84.8)	72.9 (64.8, 79.9)	88.3 (80.88, 91.9)	<0.001	74.55 (66.41, 84.23)
LY% (median (IQR))		15 (8.3, 21.8)	17.3 (11.3, 23.4)	5.6 (3.5, 10.72)	<0.001	14.9 (9.5, 21.6)
MONO% (median (IQR))		7.1 (5.2, 9.4)	7.6 (6, 9.7)	5.2 (3.25, 7.53)	<0.001	7.8 (5.85, 9.95)
Hb (median (IQR))		120 (103, 134)	122 (107, 134)	114 (91, 133.25)	0.002	118 (106, 130)
PLT (median (IQR))		164 (126, 219)	162 (125, 212)	170.5 (128.75, 233.25)	0.158	159 (119.5, 223)
NLR (median (IQR))		5.04 (3.1, 10.21)	4.16 (2.8, 7.12)	15.72 (7.61, 26.15)	<0.001	5.14 (3.01, 8.53)
PLR (median (IQR))		164.54 (106.99, 252.05)	147.25 (101.85, 218.79)	266.4 (157.35, 451.66)	<0.001	167.44 (112.5, 250.42)
SII (median (IQR))		842.34 (459.12, 1789.07)	682.08 (402.58, 1204.17)	2425.51 (1310.66, 4679.14)	<0.001	771.2 (462.32, 1696.27)
ALT (median (IQR))		30 (17, 62)	29 (16, 53)	33.5 (20, 112.25)	0.004	25 (17, 51)
AST (median (IQR))		37 (26, 65)	36 (25, 56)	50 (29, 134)	<0.001	36 (26, 58)
AchE (median (IQR))		4305 (3441, 5256)	4510 (3698, 5417)	3497.5 (2669.25, 4518.25)	<0.001	4679 (3644, 5522)
TP (mean ± SD)		65.64 ± 7.42	66.26 ± 7.08	63.56 ± 8.13	<0.001	66.76 ± 6.51
ALB Admission (median (IQR))		35.5 (32.7, 37.3)	36.1 (33.9, 37.5)	32.15 (29.37, 35.52)	<0.001	35.9 (32.7, 37.5)
ALB discharge (median (IQR))		34.9 (32.1, 37.3)	35.5 (33.1, 37.7)	32.4 (29.37, 35.23)	<0.001	35.7 (32.5, 37.7)
ALB variation (median (IQR))		0 (–1.6, 0.7)	0 (–1.4, 0.8)	0 (–1.92, 0.53)	0.579	0 (–0.3, 0)
NPAR (median (IQR))		2.13 (1.86, 2.5)	2.03 (1.79, 2.3)	2.64 (2.4, 3.02)	<0.001	2.11 (1.84, 2.39)
UA (median (IQR))		438 (325, 566)	422 (319, 539)	511.5 (363.75, 642.5)	<0.001	388 (300, 515)
Cr (median (IQR])		97 (73, 153)	91 (70, 130)	132.5 (86, 220.75)	<0.001	89 (67.5, 155.5)
BUN (median (IQR))		10.2 (7.41, 15.89)	9.37 (7.02, 13.82)	15.89 (10.5, 22.65)	<0.001	9.25 (7.04, 14.61)
K (median (IQR))		3.93 (3.53, 4.41)	3.89 (3.53, 4.34)	4.12 (3.54, 4.77)	<0.001	3.93 (3.51, 4.38)
Na (median (IQR))		138 (134, 141)	139 (135, 142)	136 (132, 140)	<0.001	140 (136, 142)
Ca (median (IQR))		1.14 (1.07, 1.2)	1.14 (1.08, 1.2)	1.1 (1.03, 1.19)	<0.001	NA
P (median (IQR))		1.21 (1.04, 1.42)	1.19 (1.04, 1.37)	1.3 (1.04, 1.79)	<0.001	1.2 (1.06, 1.42)
Hco3 (median (IQR))		23 (19.8, 26.1)	23.3 (20, 26.2)	21 (18, 26)	<0.001	23.25 (20, 27.45)
Mg (median (IQR))		0.86 (0.78, 0.96)	0.86 (0.78, 0.94)	0.88 (0.78, 1.01)	0.032	0.86 (0.79, 0.95)
CK (median (IQR))		65.00 (43.00, 105)	63 (43, 98)	75.5 (42, 168.75)	0.011	62 (39, 92)
CK-MB (median (IQR))		11.00 (6.00, 16.00)	10.00 (6.00, 15.00)	12 (6.00, 21.00)	0.017	9.00 (4.00, 15.00)
CRP (median (IQR))		15.13 (5, 40.4)	10.74 (5, 30.85)	38.2 (15.5, 84.53)	<0.001	15.67 (5, 38.58)
LCR (median (IQR))		0.07 (0.02, 0.18)	0.1 (0.03, 0.21)	0.02 (0.01, 0.06)	<0.001	0.07 (0.02, 0.17)
NT-proBNP (median (IQR))		17,100 (9790, 30,000)	15,210 (9360, 27,900)	27,710 (16,950, 30,000)	<0.001	15,639 (7882, 30,000)
cTnl (median (IQR))		0.05 (0.02, 0.12)	0.04 (0.02, 0.09)	0.11 (0.04, 0.43)	<0.001	0.04 (0.02, 0.12)
TC (median (IQR))		3.28 (2.75, 3.95)	3.29 (2.83, 3.98)	3.17 (2.4, 3.86)	0.005	3.46 (2.96, 3.97)
TG (median (IQR))		1.01 (0.77, 1.34)	0.98 (0.76, 1.32)	1.06 (0.81, 1.45)	0.026	0.96 (0.73, 1.35)
HDL-C (median (IQR))		0.97 (0.79, 1.25)	0.98 (0.8, 1.24)	0.92 (0.66, 1.28)	0.005	1.03 (0.82, 1.28)
LDL-C (median (IQR))		1.81 (1.37, 2.33)	1.83 (1.44, 2.34)	1.74 (1.19, 2.3)	0.01	1.85 (1.35, 2.47)
ApoA1 (median (IQR))		0.82 (0.68, 0.99)	0.84 (0.72, 1.00)	0.74 (0.55, 0.91)	<0.001	NA
PHR (median (IQR))		164.71 (118.8, 237.97)	162.18 (118.02, 227.38)	174.82 (124.21, 300.52)	0.005	153.57 (109.27, 208.49)
LOS (median (IQR))		9 (7.00, 14.00)	10 (7.00, 14.00)	6 (2.75, 13.00)	<0.001	9 (7.00, 13.00)
HAS (median (IQR))		0 (0, 0)	0 (0, 0)	0 (0, 4)	<0.001	0 (0, 0)

Values are n (%), mean ± SD, or median (IQR). CAD, coronary artery disease; HTN, hypertension; DM, diabetes mellitus; EN, enteral nutrition; UTI, urinary tract infection; LVEF, left ventricular ejection fraction; ICU, intensive care unit; CCU, coronary care unit; BMI, body mass index; GNRI, geriatric nutritional risk index; WBC, white blood cell; ANC, absolute neutrophil count; ALC, absolute lymphocyte count; AMC, absolute monocyte count; NEUT%, neutrophil percentage; LY%, lymphocyte percentage; MONO%, monocyte percentage; Hb, hemoglobin; PLT, platelet count; NLR, neutrophil-to-lymphocyte ratio; PLR, platelet-to-lymphocyte ratio; SII, systemic immune-inflammation index; LCR, lymphocyte-to-c reactive protein ratio; ALT, alanine aminotransferase; AST, aspartate aminotransferase; AchE, acetylcholinesterase; TP, total protein; ALB, albumin; NPAR, neutrophil percentage to albumin ratio; UA, uric acid; Cr, creatinine; BUN, blood urea nitrogen; CK, creatine kinase; CK-MB, creatine kinase-myocardial band; cTnI, cardiac troponin I; NT-proBNP, N-terminal pro-B-type natriuretic peptide; TC, total cholesterol; TG, triglyceride; HDL-C, high density lipoprotein cholesterol; LDL-C, low density lipoprotein cholesterol; ApoA1, apoprotein A1; LOS, length of stay; PHR, platelet to high density lipoprotein cholesterol ratio; HAS, human albumin supplementation; CRP, C-reactive protein. *p*-values correspond to comparisons between survival and death.

### 2.3 Model Development, Evaluation and Interpretation

In this research, 889 patients from the BMU 1st AH were divided into training and validation cohorts at a 7:3 ratio through stratified sampling, while 191 patients from BCH as the testing cohort. The training cohort, which constitutes the largest proportion of the dataset, is used for model learning and parameter optimization. The validation cohort monitors the training process to prevent overfitting and helps fine-tune model performance. Finally, a completely independent test cohort evaluates the model’s final performance after training and parameter adjustment are completed. The XGBoost model achieved higher predictive accuracy than other machine learning models in forecasting cardiovascular events based on laboratory test results [16, 17]. Given that the primary data we collected consisted of laboratory test results and that HF was either the primary or secondary cause of death in deceased patients, we choose to construct an XGBoost model to predict patient mortality. Hyperparameter tuning was performed using a systematic grid search approach via the train function from the caret package in R. The objective was to optimize the performance of the XGBoost model, with the primary evaluation metric set to the logarithmic loss (logloss) calculated through cross-validation. After an exhaustive search within the defined parameter space, the final optimal configuration was determined as follows: a learning rate of 0.002, a maximum tree depth of 2, 200 boosting rounds, a gamma value of 0, a column sampling rate of 0.6, a minimum child weight of 1, and a subsample ratio of 0.8. This specific combination of parameters was selected as it yielded the lowest cross-validated logloss, thereby providing the model with the best generalizability on the given dataset within the explored search space. Model performance was evaluated using the receiver operating characteristic (ROC) curve, calibration curve, and decision curve analysis (DCA). Additionally, metrics including accuracy, sensitivity, and specificity were calculated and reported to comprehensively assess the predictive performance of the model. To address the inherent opacity ML algorithms and improve model interpretability, Shapley Additive Explanations (SHAP) values were applied for feature importance analysis. A bar plot was generated to visualize the contribution of each feature to the model’s predictions.

### 2.4 Statistical Analysis

All statistical analyses and data visualizations were performed using R4.3.0 (R Foundation for Statistical Computing, Vienna, Austria). Continuous variables were presented as mean ± standard deviation (normally distributed data) or median (IQR) (non-normally distributed data), while categorical variables were expressed as frequencies (percentage). For between-group comparisons, Chi-square tests were used for categorical variables, and independent-sample *t*-tests (normal distribution) or nonparametric tests (non-normal distribution) were applied for continuous variables, as appropriate.

### 2.5 Laboratory Instrumentation and Assays

Complete blood count analysis was performed at BMU 1st AH (Bengbu, Anhui, China) using a Sysmex XN-9000 hematology analyzer (Sysmex Corporation, Kobe, Japan), and at BCH (Bengbu, Anhui, China) using a Mindray BC-7500 hematology analyzer (Shenzhen Mindray Bio-Medical Electronics Co., Ltd., Shenzhen, China). Biochemical testing at BMU 1st AH was conducted on a Cobas c 701 analyzer (Roche Diagnostics, Basel, Switzerland). Similarly, biochemical testing at Bengbu Central Hospital was independently performed using a Cobas c 701 analyzer of the same model (Roche Diagnostics, Basel, Switzerland).

## 3. Result

### 3.1 Baseline Characteristics of Study Population

The study flowchart is shown in Fig. 1. After applying the inclusion and exclusion criteria, clinical data from 889 patients were ultimately utilized to generate both the training set and validation set. Table 1 presents the baseline characteristics of these patients, primarily including demographic information, laboratory test results and comorbidities. Among the 889 elderly patients (median age was 78 years) with HF and malnutrition in the training and validation sets, 564 patients (63.44%) had CAD, 520 patients (58.49%) had arrhythmia, 446 patients (50.16%) presented with pulmonary infection upon admission and 437 patients (49.15%) had HTN. We employed stratified sampling for data partitioning, ensuring balanced baseline characteristics between the training and validation sets. With the addition of 191 cases from BCH for external validation, the total study population comprised 836 survivors and 244 deaths. The endpoint event rates in the training set, validation set and testing set were 22.953%, 22.932% and 20.942%, respectively.

### 3.2 Identification of Optimal Predictive Factors 

**Fig. 2.**
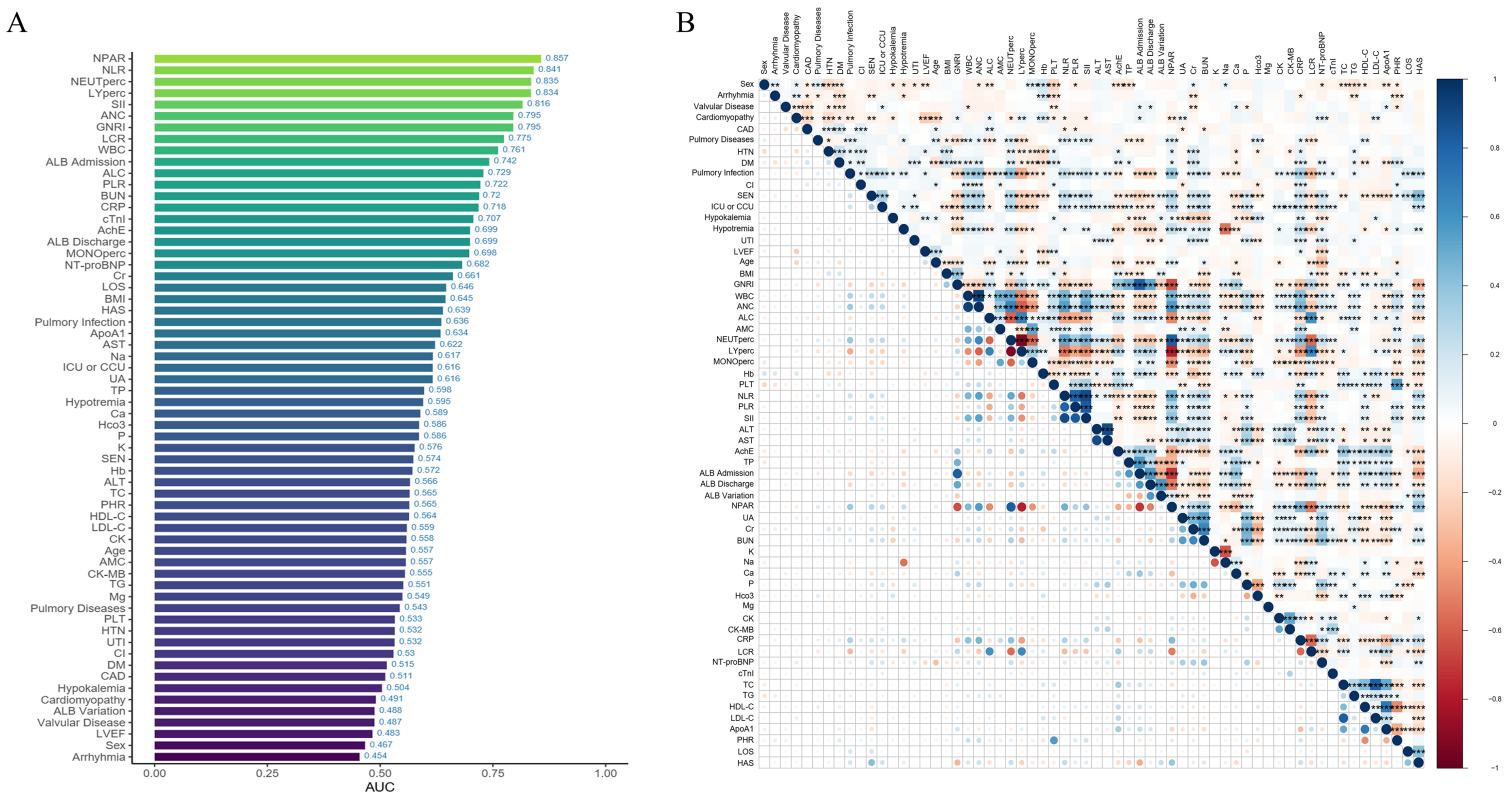
**Variable importance and correlation analysis**. (A) AUC values of each variable. (B) Heatmap of correlations among variables, with more intense colors indicating higher correlation. *, **, ***, and **** indicate statistical significance at different levels, with more asterisks representing stronger significance.

**Fig. 3.**
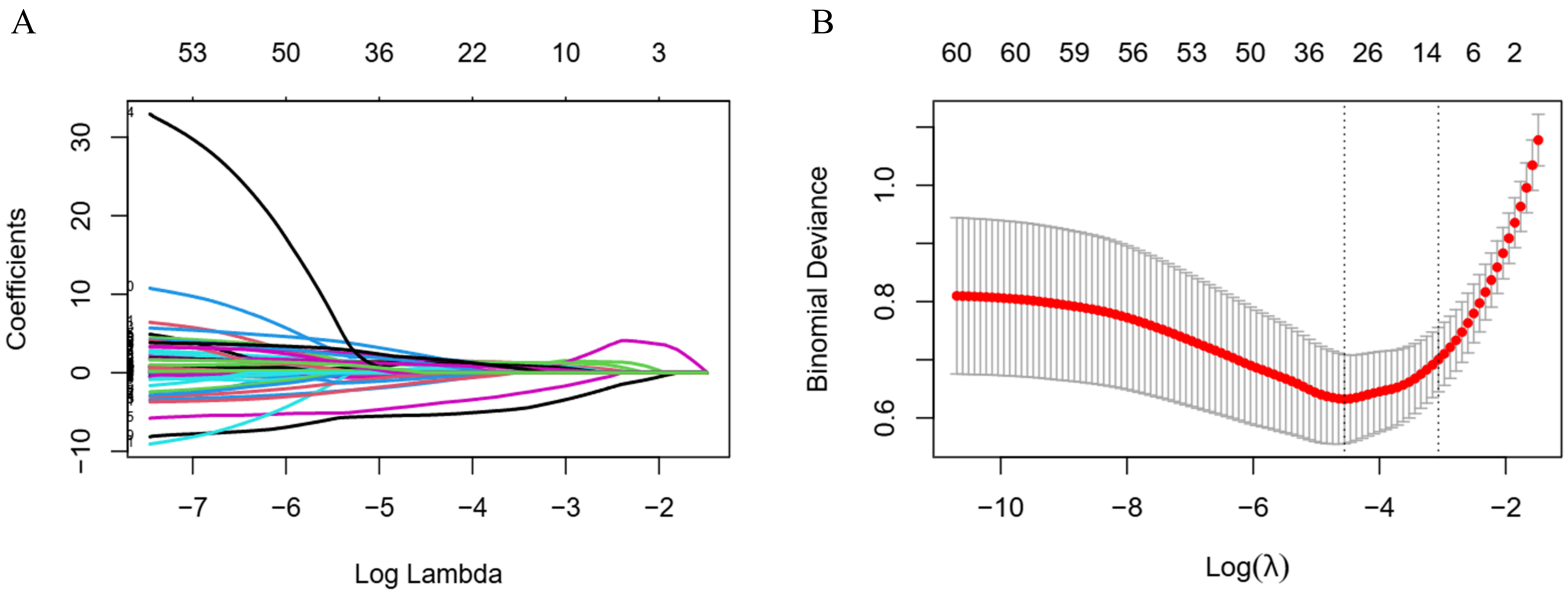
**LASSO regression for variable selection**. (A) LASSO coefficient path plot. (B) LASSO cross-validation curve, the vertical dashed lines indicate the optimal λ values where the model achieves improved predictive performance while retaining the corresponding variables. LASSO, least absolute shrinkage and selection operator.

Pairwise correlations among all 62 candidate variables were calculated using spearman methods with significance levels (*p*-values) adjusted for multiple comparisons via the Benjamini-Hochberg procedure. Results were visualized in a correlation heatmap (Fig. 2), revealing significant collinearity among multiple biomarker pairs. To mitigate multicollinearity effects on model stability, we employed iterative LASSO with 10-fold cross-validation [18]. The optimized hyperparameter λ was identified based on the bivariate deviation. Based on the optimal λ, 13 predictors are identified from the initial pool of 62 candidate variables, as seen in Fig. 3, in order: GNRI, NEUTperc, LYperc, NT-proBNP, BUN, NLR, ANC, serum potassium (K), PLR, serum phosphorus (P), PHR, Hyponatremia, and AST.

### 3.3 XGBoost Model Performance Evaluation 

**Fig. 4.**
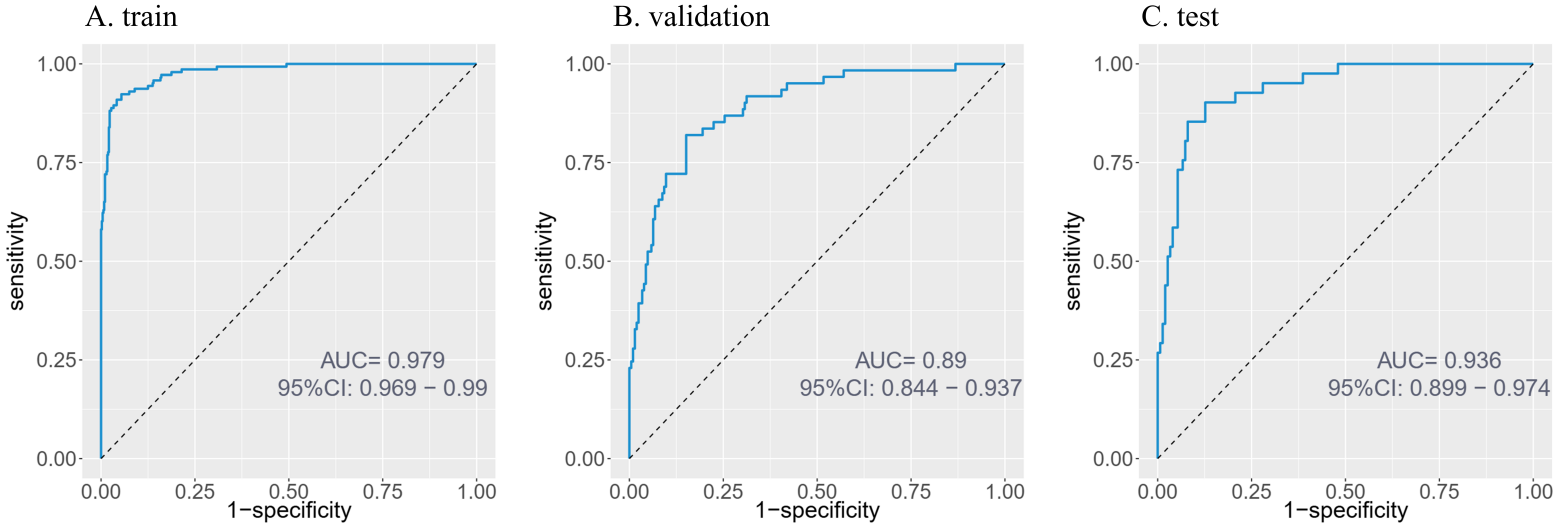
**ROC curves of the predictive model**. (A) Training set. (B) Validation set. (C) Testing set. ROC, receiver operating characteristic.

The XGBoost model demonstrated robust performance in predicting mortality among elderly patients with HF and malnutrition. Key metrics across training, validation and test datasets are summarized in Table 2 and Fig. 4. The model exhibited outstanding discriminative ability (training AUC = 0.979, testing AUC = 0.936). While specificity remained high (>94% across all sets), sensitivity dropped significantly in validation (52.4%) and test sets (58.5%). The F1-score reflected this imbalance (training: 0.877 vs testing: 0.675), suggesting potential under-detection of true mortality cases in external data.

**Table 2.** **Performance evaluation of XGBoost in the training, validation, and testing set**.

	Training	Validation	Testing
AUC	0.979 (0.969–0.990)	0.890 (0.844–0.937)	0.936 (0.899–0.974)
Sensitivity	0.923	0.524	0.585
Specificity	0.945	0.951	0.960
Accuracy	0.940	0.853	0.879
Precision	0.835	0.761	0.800
F1 score	0.877	0.621	0.675

Values in parentheses are 95% CI. XGBoost, extreme gradient boosting.

### 3.4 Interpretation of the XGBoost ML Model

**Fig. 5.**
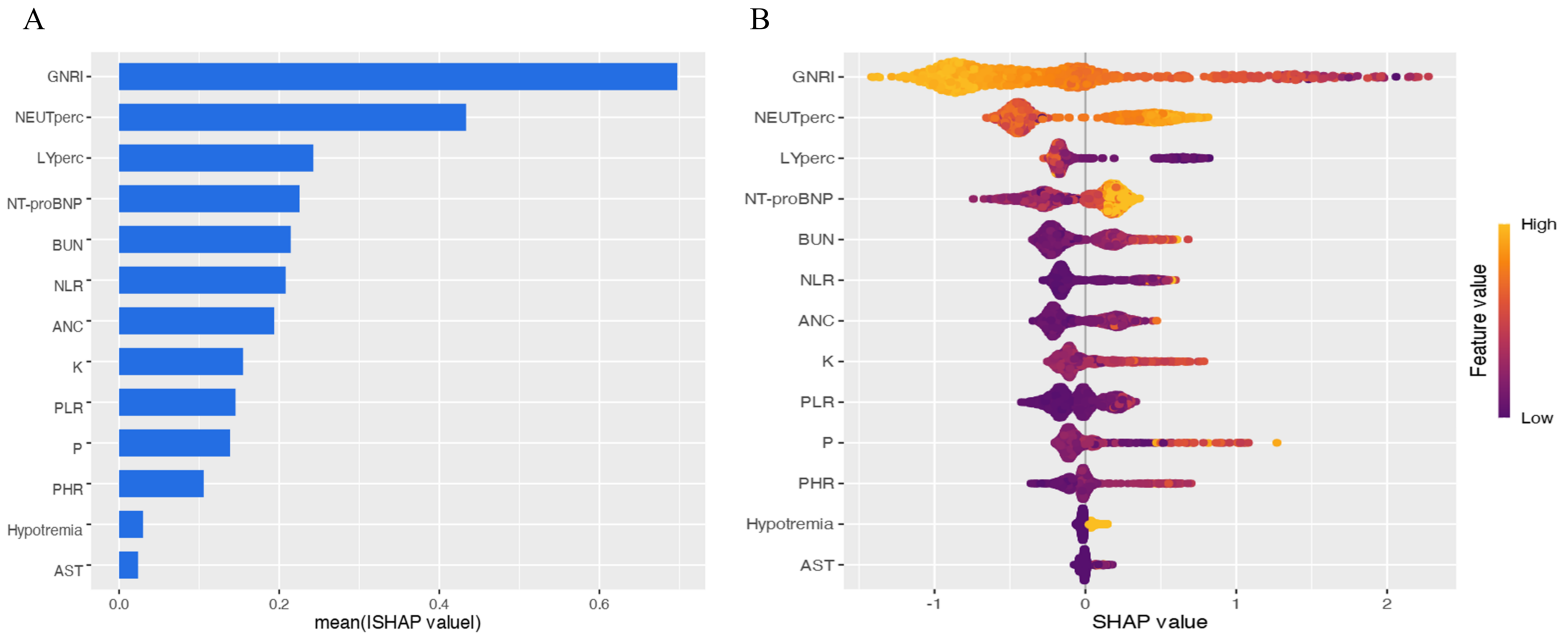
**Feature importance and impact analysis**. (A) Feature importance plot. Features are ranked by mean absolute SHAP values in descending order. (B) Beeswarm plot of significant variables, demonstrating the impact of each feature on model output. Each dot represents a patient, with color indicating feature value (yellow: higher values; purple: lower values). Increased dot dispersion suggests greater influence on predictions. SHAP, shapley additive explanation.

The SHAP summary plot demonstrates the relative contributions of each feature to the predictive model, with variables ranked by their mean absolute SHAP values in descending order: GNRI, NEUTperc, LYperc, NT-proBNP, BUN, NLR, K, PLR, P, PHR, hyponatremia, and AST. Higher SHAP values for a given feature indicate greater association with increased mortality risk. To enhance interpretability, we generated a beeswarm plot (Fig. 5) that visually distinguishes features with positive versus negative impacts on mortality prediction: yellow data points (higher feature values) generally represent risk-increasing effects, while purple (lower values) shows protective effects, with the intensity of color reflecting the magnitude of SHAP value contribution.

### 3.5 Clinical Utility: Calibration and Decision Curves

**Fig. 6.**
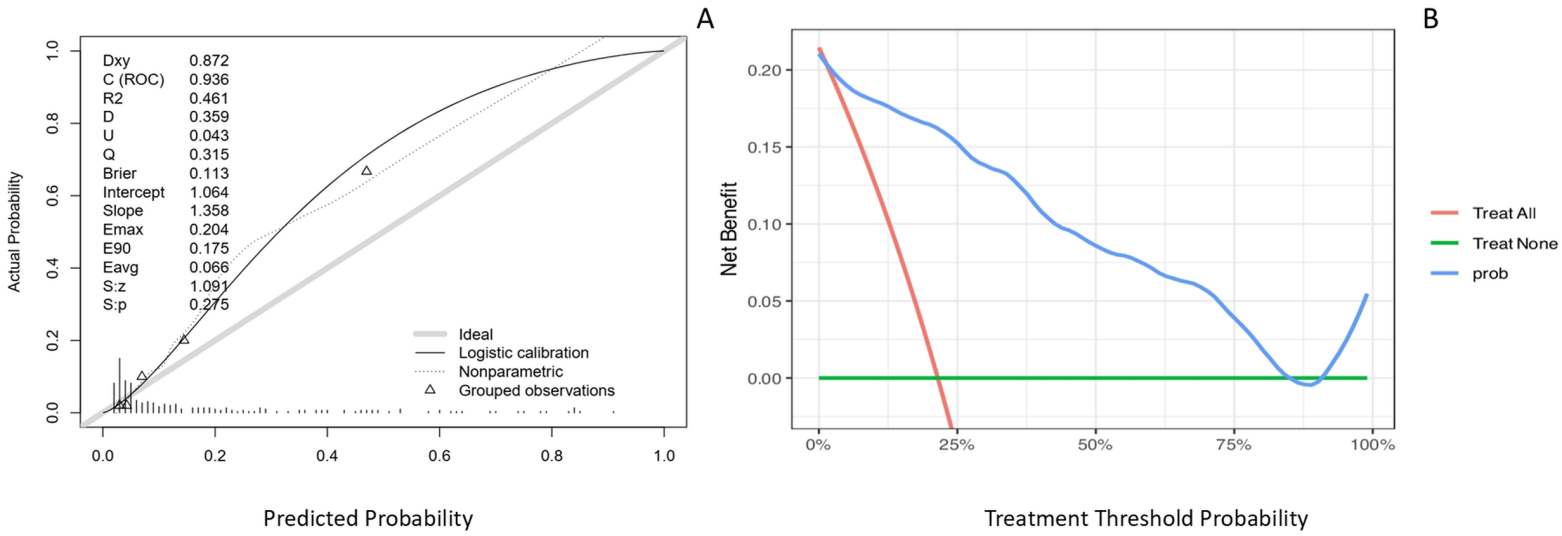
**Model calibration and clinical utility evaluation**. (A) Calibration curve plotted on the testing set to evaluate the agreement between predicted and observed probabilities. The gray solid line represents the ideal reference where predicted probability equals observed probability. The black solid line indicates the logistic calibration fit, reflecting the overall calibration trend of the model. The dotted line depicts the nonparametric calibration curve, capturing local deviations from ideal calibration. (B) Clinical decision curve based on the testing set. The x-axis is the risk threshold, and the y-axis is the benefit. The blue line represents intervening only when the model predicts risk above the threshold, the red line represents treating all patients, and the green line represents treating none. Where the blue line lies above the other two, using the model provides the greatest net benefit.

This study constructed calibration curves and clinical decision curves based on an independent test set (Fig. 6). The logistic calibration curve exhibited a non-parallel deviation, with a calibration intercept of 1.064, indicating an overall systematic underestimation of predicted probabilities relative to the actual observed probabilities. The calibration slope was 1.358, suggesting that the model was overconfident in distinguishing between high-risk and low-risk groups. The model demonstrated good discriminative ability, with a Dxy value of 0.872, reflecting its effectiveness in risk stratification between cases and non-cases. The overall prediction error, as measured by the Brier score, was 0.113, which falls within an acceptable range and was primarily attributed to calibration bias. Furthermore, the nonparametric calibration curve showed a parallel deviation, indicating consistency between the risk ranking derived from the model and the observed risks. In summary, the model exhibited satisfactory discriminative performance and interpretability, however, its suboptimal calibration limits the reliability for direct application, and further validation or calibration refinement is warranted to enhance predictive accuracy.

The decision curve analysis revealed that across a broad threshold probability range of approximately 10% to 75%, the net clinical benefit of using this model was higher than that of the two empirical strategies: treat all and treat none. These results support the model’s utility as an effective tool for clinical decision-making.

## 4. Discussion 

The principal findings of this investigation reveal a critically high in-hospital mortality rate of 22.6% among elderly patients afflicted with the dual burden of HF and malnutrition. The XGBoost model achieves remarkable predictive accuracy, as illustrated in Fig. 4 (train AUC: 0.979; validation AUC: 0.890; test AUC: 0.936). And as shown in Fig. 5, the application of SHAP analysis for interpretable machine learning yielded a nuanced hierarchy of predictive features. GNRI emerged as the paramount predictor, followed closely by a constellation of inflammatory and cellular stress markers, including NEUTperc, LYperc, and NLR. This feature importance profile compellingly suggests that in-hospital mortality in this vulnerable cohort is driven by a complex interplay of nutritional depletion, systemic inflammation, and neurohormonal activation.

GNRI emerged as the most influential predictor within our predictive model. This finding aligns with previous studies demonstrating that GNRI serves as an independent risk factor for adverse prognosis in HF populations [2]. The strong predictive power of GNRI stems from its role as more than just a nutritional screening tool. In the advanced stages of heart failure, patients often experience a vicious cycle involving systemic inflammation, autonomic dysfunction, and cachexia, clinically manifested as anorexia and progressive muscle wasting [2]. As a composite measure of serum albumin and body weight, GNRI is more sensitive than single-dimensional nutritional indices such as BMI in identifying this state of heightened inflammation and hypermetabolism. Therefore, in elderly heart failure patients, GNRI serves not merely as an assessment of nutritional status but, more importantly, as an integrative early warning signal for developing or impending cardiac cachexia. A lower GNRI score may thus indicate poorer nutritional status, a higher likelihood of cachexia, and an elevated risk of mortality.

The high contribution of inflammatory cell components (neutrophil and lymphocyte percentages) and the composite index NLR in the SHAP analysis is a key finding. This pattern collectively underscores the strong association of systemic inflammation with mortality risk. Previous research has shown that in the context of HF-induced myocardial injury, the immune cell recruitment cascade-primarily mediated by neutrophils and lymphocytes-plays a critical role in coordinating tissue repair [4]. The strong predictive value of NLR further supports that immune dysregulation, as reflected by this index, is a salient feature of high-risk patients. Recent studies have increasingly explored the association between novel composite inflammatory markers and adverse HF outcomes, revealing that elevated NLR correlates with more severe myocardial damage and worse prognosis [10, 11]. Our findings further validate that NLR remains a robust prognostic indicator even in elderly malnourished HF patients. Notably, while NPAR demonstrated the highest predictive power in univariate analysis (AUC = 0.857), it was not retained in the final LASSO regression model. This is consistent with LASSO’s regularization property, which tends to retain a single, most representative variable from a group of correlated predictors to avoid redundancy. The selection of GNRI, which shares the albumin component with NPAR, likely reflects this statistical parsimony, rather than the independent prognostic irrelevance of NPAR. Its high univariate AUC underscores NPAR’s potential clinical utility as a rapid screening tool, warranting further investigation.

Consistent with its well-established prognostic value in HF, NT-proBNP emerged as a strong predictor of mortality in our model [1]. Similarly, hyponatremia demonstrated significant association with increased mortality risk among elderly HF patients with malnutrition, aligning with prior epidemiological observations. The predictive importance of elevated blood urea nitrogen levels likely reflects, in pathophysiological terms, the cascade of HF-induced renal hypoperfusion and subsequent prerenal injury [15]. The presence of these indicators in the model’s accurate capture of the core cardiorenal axis in HF.

Furthermore, electrolyte disturbances are frequently observed in patients with HF and are often exacerbated by diuretic therapy. Previous studies have frequently demonstrated a U-shaped relationship between serum potassium levels and HF outcomes, wherein both hypokalemia and hyperkalemia are associated with increased mortality risk [19, 20]. While our findings similarly indicate that both low and high potassium levels are associated with mortality risk, the predictive model particularly identified hyperkalemia as the more prominent risk indicator in this specific cohort. This discrepancy could be influenced by several factors: (a) population characteristics: our study specifically focused on elderly malnourished HF patients, who may exhibit distinct electrolyte homeostasis compared to general HF populations. (b) nutritional interactions: the interplay between malnutrition and electrolyte imbalances may amplify the prognostic significance of hyperkalemia.

## 5. Limitations

This study has several limitations. Its retrospective design may introduce selection bias, information bias, and unmeasured confounding. Furthermore, the generalizability of the findings is constrained by the exclusive use of data from two tertiary centers in China and the restriction of the primary outcome to in-hospital mortality. The model’s performance and clinical applicability may also be affected by the lack of detailed treatment data and by potential class imbalance in the dataset, which were not explicitly addressed. Additionally, while in-hospital mortality is a clinically significant endpoint, it does not capture other vital long-term outcomes in heart failure management, such as post-discharge survival, readmissions, or quality of life. Finally, the model exhibited a significant decline in sensitivity on both the validation and test sets, indicating a high missed detection rate on new data, which aligns with the systematic underestimation suggested by the calibration results. This outcome may be attributed to shifts in sample feature distributions and potential overfitting of the model to certain specific noise in the positive samples within the training set. Further simplification and revalidation of the model will require datasets with more positive samples and greater diversity of sources. Although the model demonstrates high discriminative ability and good clinical applicability, its reliability for direct application is limited due to the missed detection of positive cases.

Given these limitations, future research should aim to collect prospective, multicenter cohorts with detailed treatment and long-term follow-up data, and explore personalized prediction models for different patient subgroups to facilitate more precise risk stratification and management.

## 6. Conclusion

Through rigorous internal and external validation, the XGBoost prediction model developed in this study demonstrated good discriminative ability in identifying the mortality risk of elderly heart failure patients with malnutrition. Although the model’s calibration performance in the test set requires further improvement, decision curve analysis indicated its practical value in clinical risk stratification. SHAP analysis further clarified the key risk factors influencing patient mortality, providing a reference for early screening and targeted interventions.

## Data Availability

The data supporting this study are not publicly available because they contain information that could compromise patient privacy under the terms of the ethical approval. Interested researchers may contact the corresponding author to discuss potential data access, subject to institutional policies and ethical requirements.

## Abbreviations

CAD, coronary artery disease; DCA, decision curve analysis; DM, diabetes mellitus; GNRI, geriatric nutritional risk index; HTN, hypertension; HF, heart failure; LASSO, least absolute shrinkage and selection operator; LCR, lymphocyte to C-reactive protein ratio; LOS, length of hospitalization; ML, machine learning; NLR, neutrophil to lymphocyte ratio; NPAR, neutrophil percentage to albumin ratio; PLR, platelet to lymphocyte ratio; PHR, platelet to high density lipoprotein cholesterol ratio; ROC, receiver operating characteristic; SHAP, shapley additive explanations; SII, systemic immune inflammation index; UTI, urinary tract infection; VHD, valvular heart disease; XGBoost, extreme gradient boosting.
